# Unraveling seizure interruptions: Excitability dynamics in spike-wave activity

**DOI:** 10.1016/j.ibneur.2026.05.005

**Published:** 2026-05-22

**Authors:** Gilles van Luijtelaar, Bob de Ruijter

**Affiliations:** a Donders Centre for Cognition, Radboud University, Nijmegen, the Netherlands; b Univé, Zwolle, the Netherlands

**Keywords:** Absence epilepsy, Cortical focus, Cortex, EEPs, Excitability, Interruption, Interval, Stimulation, Spike, SWD, WAG/Rij rat, Wave

## Abstract

Neuronal activity during a spike–wave complex (SWC) consists of highly synchronized firing of neuronal populations followed by a period of neuronal silence. It may manifest as an interictal spike or repetitive. Cortical excitability during the ascending and descending slope of the spike and during the three wave components of Spike-Wave discharges is examined in a well-established genetic rodent model of absence epilepsy using single-pulse electrical stimulation. The probability of interrupting ongoing SWDs and the electrical evoked response were assessed. Single-pulse stimulation could interrupt SWDs, primarily in the first second of an SWD, but also later, irrespective of whether stimulation occurred during the spike or wave. The amplitude of the P1 recorded from the somatosensory cortex was significantly higher during the descending phase of the spike than during the spike’s ascending phase and the wave components. During the wave component, P1 amplitudes increased toward the end. The P1 made during non-REM sleep resembled those of the wave. The differences between the first second and later might be due to differences in coupling between cortex and thalamus and to the dynamics of the intraspike frequency, the differences between the two phases of the spike to the readiness and possibility to fire, while the inhibition during the wave is due to hyperpolarization. Further, the possibility of interrupting an SWD by a single pulse is not related to the changes in cortical excitability, but seems caused by an interruption of the oscillatory cortico-thalamo-cortical network driving SWDs.

## Highlights

• Dynamics in both Intraspike and Intrawave excitability were detected in WAG/Rij rats.
• Two measures of excitability across a spike-wave complex (SWC) show opposite results.
• Evoked peak-peak amplitudes don’t correctly measure the dynamics of excitability of a SWC.
• Interruptions of SWD are easier in the first second of an SWD than later.
• The amount of inhibition during the wave is comparable to that during non-REM sleep.

## Introduction

Absence epilepsy is a neurological disease that affects approximately 50 million people worldwide, and approximately 2–8 per 100,000 children under the age of 16 develop absence epilepsy each year (Kwan and Brodie, 2000, Loiseau et al., 1990, Sander and Shorvon, 1996). Though absence epilepsy is often considered to disappear after puberty, absences may persist in 7–81% of the juveniles and adults (Panayiotopoulos et al., 1992). In almost one-third of the cases, absence epilepsy will develop into a more severe epilepsy or a mixture of absence and convulsive epilepsy when reaching adolescence (Karbowski, 1985). The seizures characterizing absence epilepsy show two hallmarks: an abrupt, brief impairment of awareness and reactivity and a concomitant highly typical electroencephalographic (EEG) pattern called spike-wave discharges (SWDs) (van Luijtelaar and Sitnikova, 2006). The SWDs appear generalized and bilateral symmetrical over the cortex, have a frequency of 3–4 Hz, with a sudden onset and offset and a duration varying between 2 and 60 s (Panayiotopoulos, 2001).

Throughout the years, several theories were proposed to reveal the possible mechanisms of absence seizures (Meeren et al., 2005; Avoli et al., 2012), and although there are fundamental differences regarding to the site of origin of SWDs, all authors describe crucial roles for both cortex and thalamus interconnected via ascending and descending pathways (Penfield and Jasper, 1954, Gloor, 1968, Gloor, 1969, Bancaud, 1969, Niedermeyer, 1972, Lüders et al., 1984, Buzsáki, 1991, Meeren et al., 2002, Polack et al., 2007, Avoli, 2012). Altered fMRI, CBV, CBF, LFP, and MUA signals during SWDs support the hypothesis of the crucial roles for cortex and thalamus in absence epilepsy (Mishra et al., 2011). Meeren et al., 2002, Meeren et al., 2005 proposed the Cortical Focus theory based on the observation that in the WAG/Rij absence model, the perioral region of the somatosensory cortex was consistently leading other cortical areas and thalamus during the initial phase of the generalized SWDs, while later, the interrelationship was more unpredictable. The cortical focal origin was confirmed in the GAERS absence model, which closely mimics the properties of the WAG/Rij strain. Elevated neural activity in the form of highly excitable cells was found in the deeper layers (V/VI) of the facial somatosensory cortex (Polack et al., 2007, Depaulis et al., 2015). Emerging evidence, including time- frequency and non-linear coupling techniques, indicated that the SWDs in rodent models as well as in children with absence seizures represent a continuous changing interplay between cortico-cortical, cortico-thalamo-cortical and intrathalamic loops, modulated by cerebellum, basal ganglia, and hippocampus (Crunelli et al., 2020, Kros et al., 2015, Lüttjohann and van Luijtelaar, 2015, Ossenblok et al., 2019, Snead, 1995, Sysoeva et al., 2014, Sysoeva et al., 2016, Terlau et al., 2020, Tolmacheva and van Luijtelaar, 2007, Medvedeva et al., 2020). A diminished inhibition and increased excitation has been found in cortical somatosensory cells in WAG/Rij rats (D’Antuono et al., 2006, Luhmann et al., 1995, Merlo et al., 2007) and this has been modelled successfully (Sargsyan et al., 2007, Medvedeva et al., 2020), suggesting that proper functioning of the cortex requires a good inhibitory-excitatory balance, otherwise pathological oscillations in the form of spike-and waves may occur.

The neurophysiology underlying the characteristic of EEG patterns has been described: the spike-component of the oscillatory SWD cycle, as measured in the accepted and validated genetic rat models and earlier in feline absence seizure models (Kostopoulos, 2000, Depaulis and van Luijtelaar, 2005), is systematically associated with synchronized membrane depolarization and action potential firing which show bursting activity of single unit and multiple units in cortical (Polack et al., 2007, Polack et al., 2009, Depaulis et al., 2015) and thalamic neurons (Inoue et al., 1993, Kandel and Buzsáki, 1997, Pinault, 2003). Complementary, multiple unit activity is spike-locked in the ascending part of the spike in layers II to VI and absent during the descending part of the spike and during the wave-component (Inoue et al., 1993, Kandel and Buzsáki, 1997, Terlau et al., 2020).

The synchronized depolarization of neurons during the spike is likely caused by underlying glutamatergic interactions of pyramidal neurons (Chipaux et al., 2011), while layer VI cortico-thalamic neurons are probably responsible for the diffuse spreading towards the thalamus as their rhythmic firing usually precedes the thalamic, relay, and reticular discharges (Pinault, 2003).

On the contrary, the wave-component is systematically associated with neural silence, membrane hyperpolarization, and it is described as a phase of transient interruption of tonic excitatory synaptic firing (Inoue et al., 1993, Kandel and Buzsáki, 1997, Pinault, 2003, Polack et al., 2007, Polack et al., 2009, Chipaux et al., 2011, Depaulis et al., 2015). Latest findings argue for a more intricate functioning neuronal network underlying both components, such as fluctuations of membrane potentials that imply an additional inhibitory involvement during the spike-component (Depaulis et al., 2015), and pyramidal cells that exhibit depolarization during the wave-component boosting and preserving the rhythmicity of pyramidal neuronal activities (He et al., 2014). Consistent with these cellular dynamics, a combined whisker stimulation and intracellular recording study in sedated GAERS rats revealed a smaller amplitude of the first component of the sensory-evoked potential and a lower firing probability during the spike than during the wave, following a 50-ms whisker stimulus (Williams et al., 2020). Building on these findings, the present study applies electrical cortical stimulation near the focal zone to probe local cortical excitability by analyzing evoked responses during the ascending and descending phases of the spike, as well as during three distinct phases of the wave component of SWDs.

Electrical cortical stimulation has also been shown to interrupt or prevent SWDs in genetic rat models of absence epilepsy (van Heukelum et al., 2016, van Luijtelaar et al., 2016, Maksimenko et al., 2017; Budde et al., 2022). However, it remains unknown whether specific phases of the SWD cycle are more susceptible to interruption by a single electrical pulse. This question constitutes a second objective of the present study.

A third objective is to determine whether cortical excitability differs between the first second following SWD onset and later phases, given the evolving dynamics of interspike frequency and cortico-cortical and cortico-thalamo-cortical interactions (Bosnyakova et al., 2007, Dolinina et al., 2022, Meeren et al., 2002, Sysoeva et al., 2016). Excitability during non-REM sleep is included as a reference condition to aid interpretation of the excitation–inhibition balance during the spikes and waves.

We hypothesize that stimulation during the spike component will have limited effects, as the network is already engaged in highly synchronized excitatory activity followed by a refractory period, leaving relatively few neurons available for recruitment. Whether excitability differs between the ascending and descending phases of the spike has not yet been investigated, but it is quite possible that during the descending phase, cortical excitatory neurons are released from firing, the membrane potential might still be depolarized, and cortical cells might be available for firing again as a consequence of stimulation. Stimulation during the wave component may partially overcome neuronal hyperpolarization and induce subthreshold or suprathreshold depolarization, although the magnitude of the response is expected to be limited.

Finally, we expect that stimulation during the first second after SWD onset will evoke larger evoked potentials, as the network is not yet fully synchronized and a larger neuronal population may be recruited. Accordingly, we hypothesize that SWDs are more readily interrupted during this early phase than during later, fully established stages.

## Animals, materials, and methods

### Animal housing

Adult male WAG/Rij rats, born and raised at the laboratory of the department of Biological Psychology of the Donders Centre for Cognition, Radboud University, Nijmegen, the Netherlands, served as subjects. The rats were approximately 8 months old and had a mean body weight of 375 g (range 326–401). Standard laboratory housing conditions were maintained as the rats were socially housed in couples in Macrolon type III cages until surgery; from then on, they were single housed, including cage enrichment and ad libitum access to food and water. A reversed 12:12 light/dark cycle was applied, with the light-off period starting at 08.30 a.m.; it allowed us to perform the experiments in the active period of the rats. The research project was approved by the Ethics Committee on Animal Experimentation of Radboud University, Nijmegen (RU-DEC).

### Stereotactic surgery: anaesthetics and electrode implantation

Implantation of stimulation and recording electrodes was done under isoflurane anaesthesia. The skull was shaved and rats were placed into a David Kopf stereotaxic instrument. Body temperature was kept constant with a heating pad. The depth of the anaesthesia was regularly checked by squeezing the feet of the rats. Methylcellulose eye drops (5 mg/ml, Théa Pharma NV) were applied to prevent eye dehydration previously to any surgical action, the analgesic Rimadyl (s.c. 0.008 ml/100 g body weight; Bela Pharm GmbH & Co. KG, Germany) was injected for pain prevention, and Atropine (i.m. 0.05 ml 1 mg/ml, Eurovet Animal Health BV, the Netherlands) was used as muscle relaxant. The skull was disinfected with iodine (100 mg/ml povidone-iodine; Meda Pharma BV), and lidocaine (lidocaine HCl 20 mg/ml and adrenaline as tartrate 10 μg/ml; Eurovet Animal Health BV) were used as a local analgesics. Rimadyl was also given 24 h and 48 h after surgery as post-surgical analgesic in the same volume and concentration as the day before.

Four electrodes of two tripolar electrode sets (Plastic-One ms333/2 A) were implanted in the right hemisphere, the ground and reference electrode were located bilateral above the cerebellum. The first recording electrode was aimed at the somatosensory cortex (S1) (AP: - 1.5, L: - 3.0), the second one in the Ventro Posterior Medial (VPM) nucleus of the thalamus (AP: −3.5, L: +2.8, H: −6.2). The two tips of the stimulation electrodes were aimed at the somatosensory cortex near the peri-oral region in layer V and VI, for the cathode we used AP: - 2.0, L: + 4.2, H: −4.52) and for the anode barrel field region (coordinates AP: - 3.0, L: + 4.6, H: 3.0). Depth coordinates were measured from the skull (Paxinos and Watson's rat brain atlas, 2007). The electrode sets were attached to the skull with dental cement (Simplex Rapid Power, Associated Dental Products Ltd Kemdent Works) to form a solid smooth cap embedding also three attachment screws. The location of the stimulation electrodes was based on the initiation zone of SWD as indicated by Meeren et al. (2002) in the same model and Polack et al. (2007) in GAERS. Considering the interindividual variation in the location of the focal zone, the two stimulation electrodes should cover a relatively large area. Next, the animals were allowed a two-week recovery period.

### Definition of spike–wave intervals

**Fig. 1:**
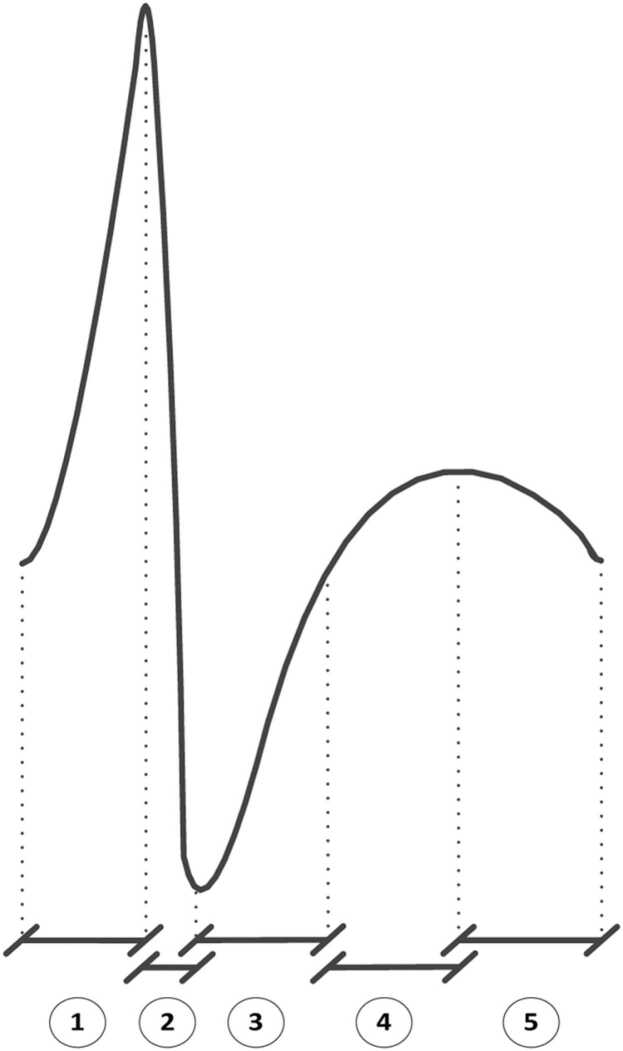
An oscillatory spike-wave cycle was divided in five intervals 1.) ascending part of the spike, 2.) descending part of the spike, 3.) and 4.) ascending part of the wave, and 5.) descending part of the wave.

Five distinct intervals of the spike–wave complex were defined (see Fig. 1): (1) the ascending phase of the spike; given an interspike interval of 8 Hz (Drinkenburg et al., 1993), it lasts about 27 msec. (2) the descending phase of the spike, lasting about 11 msec. (3) the first half of the ascending phase of the wave, lasting about 28 msec. (4) the second half of the ascending phase of the wave, lasting about 28 msec, and (5) the descending phase of the wave, lasting about 31 msec.

### Experimental set-up

Rats were housed individually in Plexiglas recording cages (20 × 35 × 25 cm) and connected via flexible recording leads to a swivel contact, allowing unrestricted movement during EEG recording and stimulation.

Two separate personal computers were used. The first PC was responsible for data acquisition, monitoring, and storage of the cortical and thalamic EEG signals, movement-related signals, detected spike events, and the onsets and offsets of both programmable delays as well as the stimulator output. Data acquisition was performed using WinDaq software (DATAQ Instruments Inc.).

A second PC ran a real-time spike–wave discharge (SWD) detection algorithm (Ovchinnikov et al., 2010) and controlled the electrical stimulator (TD10075; TSG, Donders Institute, Radboud University).

EEG signals were amplified using a physiological amplifier (TSG, Donders Institute, Radboud University), band-pass filtered between 1 and 1000 Hz, and additionally filtered with a 50 Hz notch filter. Signals were digitized at a sampling rate of 1024 Hz.

Stimulation pulses were generated using a programmable stimulus generator (TSG, Radboud University), interfaced with the TD10075 stimulation control software. The timing of stimulation delivery following spike detection was controlled by a programmable hardware-based modular delay system (TSG, Radboud University). This system allowed precise temporal control of stimulation relative to detected spike events.

The first programmable delay enabled stimulation to be delivered between 1 and 100 ms after automatic spike detection. A second delay, adjustable between 200 and 5000 ms, prevented the delivery of subsequent stimulation pulses within a predefined refractory interval. This design ensured that the effects of a single stimulation pulse could be evaluated without interference from additional pulses.

Animal movement was monitored using a Passive Infrared Registration (PIR) system (LuNAR PR, Risco Group), which provided a quantitative measure of gross body movements (van Luijtelaar and van Oijen, 2020).

### Closed-loop system

A closed-loop system was employed for the detection of spike activity and the contingent delivery of cortical stimulation. The cortical EEG signal served as the input to the system, while the SWD detection software and the stimulation control program operated concurrently. The SWD detection algorithm provided trigger signals to a modular timing system, which precisely controlled the timing of the electrical stimulation pulses generated by the stimulator. Subsequently, the stimulator delivered these pulses to the targeted focal cortical area, thereby completing the closed-loop configuration.

**Fig. 2:**
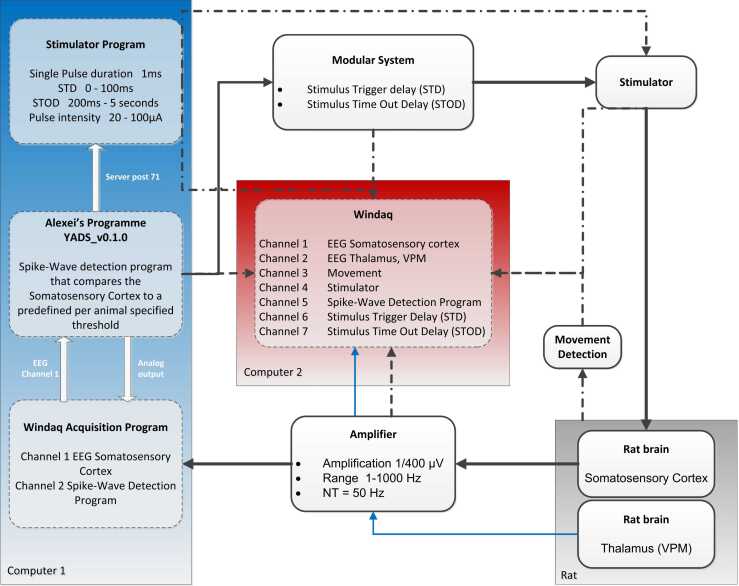
Fig. 2**.** Experimental set-up. Black line: closed-loop system (physical connection). The red box represents the monitoring and data storage by the WINDAQ system of the cortical and thalamic EEG, the signal from the infrared movement detector (PIR), the timing of the stimulators on- and offset, the timing of the automated detection by the SWD detection Program/YADS_v0.1.0, the on- and offset of the stimulation trigger delay (STD, 0, 40 or 100 msec), and the on- and offset of the stimulation time-out delay (STOD, varying from 200 msec to 5 s.; it allowed us to stimulate after the first second). The thalamic EEG recording (blue line) was used only to monitor whether the stimulation also affected the presence of SWDs in the thalamus. The blue box shows the programs that are simultaneously running to detect spikes and the timing of the on- and offset of electrical stimulation. The black dotted lines indicate the interactions of the programmed systems.

A schematic overview of the instrumentation and signal flow of the experimental setup is shown in Fig. 2.

#### Experimental sessions

Animals were habituated to the EEG recording setup for 15 h before the start of the experimental procedures. Subsequently, a one-hour stimulation-free baseline recording session was conducted to determine individual spike detection thresholds.

Three experimental stimulation sessions were then performed over the course of the following two weeks. Each session lasted three hours. During all sessions, single monophasic stimulation pulses with a duration of 1 ms were delivered.

The stimulation delay (STD) was systematically varied within each session: during the first hour the STD was set to 1 ms, during the second hour to 40 ms, and during the third hour to 100 ms. This design allowed stimulation to be applied at different temporal phases of the spike–wave complex.

Three stimulation intensities (20, 60, and 100 µA) were used. Intensities varied between sessions and were always delivered in ascending order to minimize potential carry-over effects from preceding stimulation conditions.

Toward the end of the final session, additional stimulation pulses were delivered manually during periods in which the EEG displayed clear, large-amplitude delta waves. These periods corresponded to non–rapid eye movement (non-REM) sleep and served as a control condition. During these manually triggered stimulations, the animals were immobile.

Six rats completed the study and were subsequently perfused for histological verification of electrode placement. Animals were deeply anesthetized using an intraperitoneal injection of ketamine–xylazine and transcardially perfused with a fixative containing formaldehyde and 2% potassium ferrocyanide.

Brains were post-fixed in a 30% sucrose solution and subsequently sectioned coronally at 100 µm thickness using a freezing microtome. Sections were mounted onto gelatin-coated glass slides, stained with Cresyl Violet, and coverslipped using Entellan mounting medium.

Electrode locations were then reconstructed and verified histologically; for the results see Supplementary Table S1. Four of the six EEG recording electrode tips were located within the somatosensory cortex, whereas two could not be reliably identified. All thalamic EEG electrodes were confirmed to be located within the ventral thalamic nuclei. Stimulation electrodes were located in subgranular layers of the somatosensory cortex or in adjacent structures, including the head of the caudate–putamen, the corpus callosum, or the hippocampus.

#### Data analysis

##### Interval and Phase-related susceptibility to interruption of spike-wave discharges

The intrinsic temporal variability in spike detection by the real-time detection algorithm, combined with the programmable delay settings, resulted in stimulation delivery across all five predefined intervals of the spike–wave cycle and after the first second. All stimulation events and the number of interrupted SWDs were analyzed for each rat and each stimulation intensity. Events were classified by BdR according to their temporal position relative to the peak of the SWD recorded in the cortical EEG. The classification implied that stimulation events were classified as occurring during either the ascending or descending phase of the spike, or during one of three wave-related intervals: two located on the ascending phase of the wave and one on the descending phase. In addition, interruptions were categorized as *early* or *late*, depending on whether they occurred within the first second of the SWD or after the first second, respectively. The early SWD interruptions were, in many cases, preceded by up to four full spike-wave cycles to make sure that a genuine SWD was occurring and detected.

To qualify as a valid interruption, the following criteria had to be met:

1. At least one complete and unambiguous spike–wave complex had to precede the stimulation, ensuring that stimulation occurred during an ongoing SWD. This spike–wave complex had to resemble the morphology of the spontaneous SWDs observed during baseline recordings in the same animal.
2. The interruption had to occur immediately after stimulation (within 100 ms), followed by EEG desynchronization without the presence of small-amplitude SWD-like events or isolated spikes for a minimum duration of 1 s.
3. Trials were excluded if a second stimulation pulse occurred within 1 s following the first pulse.

Quantification of both early and late SWD interruptions was performed per interval (1–5), per stimulation intensity (20, 60, and 100 µA), and per subject. When comparing early and late interruptions, it should be noted that interruptions occurring later than 1 s after SWD onset may not reflect the first stimulation attempt. In these cases, the animals were likely stimulated at least once previously without an observable interruption.

In addition, interruption percentages per interval were calculated to correct for potential unequal distributions of stimulation events across the five SWD intervals. Such inequality was expected, as stimulation was automatically triggered by the SWD detection algorithm, which required a variable amount of time to detect a genuine spike–wave complex.

In cases of uncertainty regarding the exact stimulation interval, the spike–wave complex in question was compared with the immediately preceding spike–wave complex. This comparison was considered the most reliable method for determining the precise stimulation interval.

### Impact of stimulation on spike-wave discharges

The SWD detection program (Ovchinnikov et al., 2010) was slightly adapted in the sense that the criterion that a SWD should last minimally one second was dropped. Now it recognizes virtually all (99.9%) of the SW complexes during the sixty hours of experimental sessions. Approximately 83% of the detected spike-wave complexes were recognized within the first 500 ms, the others within the next 500 msec. The number and duration of SWDs per hour were determined for all four experimental sessions (baseline-20–60–100 μA) to reveal the impact of stimulation on these variables. This allowed us to verify whether stimulation perse was able to evoke SWD or Afterdischarges, mimicking SWDs (Lüttjohann et al., 2011).

#### Electrical evoked potentials (EEP)

For each rat, ten stimulation trials per interval and per stimulation intensity in the early and late phase of a SWD were selected of the SWD. This selection was performed blindly with respect to the experimental condition. The stimulation-evoked responses were then analyzed, and grand-average waveforms were computed.

It should be noted that responses (EEPs) recorded as local field potentials within the brain show substantially less inter-trial variability than scalp-recorded evoked potentials. As a result, reliable evoked responses can be obtained by averaging a relatively small number of trials; in the present study, averaging ten responses per condition was sufficient.

BrainVision Analyzer 2.0 (Brain Products GmbH) was used for all analyses of the EEPs. They were computed per SW complex interval, in the early (< 1 s after SWD onset) and late phase of the SWD (> 1 s after SWD onset), per stimulation intensity, and—during the third session—also during non-REM sleep.

The extracted EEG epochs started 20 ms before the stimulation pulse and continued for 100 ms thereafter. Grand-average waveforms revealed, in addition to the initial stimulation artifact, a sequence of identifiable components consisting of a positive peak (P1), followed by a negative peak (N1), and a subsequent positive peak (P2). The following dependent variables were extracted: the amplitudes of P1, N1, and P2; the peak-to-peak amplitudes from the stimulation artifact to P1 (AP1), from P1 to N1 (P1–N1), and from N1 to P2 (N1–P2); and the latencies of the P1, N1, and P2 components.

### Statistical analysis

The susceptibility for the interruptions as well as the EEP characteristics (nine dependent variables: the amplitudes and latencies of P1, N1, P2, and the amplitudes of artP1, P1N1, and N1P2) were analysed with a two-factor (intensity and interval as within-subject factors) repeated measures ANOVA, followed by pairwise comparisons or by paired sample Students *t*-tests. Effect sizes were expressed by partial eta squared. Interaction effects were disentangled by one-factor repeated measures ANOVAs as post-hoc tests, again followed by paired sample *t*-tests, if appropriate. The Bonferroni correction was used in all statistical tests to correct for the use of multiple comparisons. Differences between the examined groups were considered significant when p < .05. Unreported analyses did not show significant effects with the post-hoc tests.

The EEPs made during NREM sleep on the one side and those made during each of the five intervals of the 100 μA condition were compared with a repeated measures ANOVA with interval (six levels: intervals 1–2–3–4–5-and non-REM sleep) as “within subjects factor”. The amplitudes and latencies of P1, N1, and P2, and the amplitudes of artP1, P1N1, and N1P2 were the dependent variables for this comparison. The phase effect (early vs. late) was analysed together with interval (five levels). Both factors were “within-subjects” factors. Again, the amplitudes and latencies of P1, N1, and P2 and the amplitudes of artP1, P1N1, and N1P2 were the dependent variables. This analyses was done for the 100 μA stimulation intensity only. SPSS Version 26 was used for all statistical analyses.

## Results

### SWD interruptions

SWDs could be interrupted by a single stimulation pulse both during the first second of an SWD (*early interruptions*) and at later time points (*late interruptions*). Interruptions occurred in all rats, at all three stimulation intensities, and across all five examined intervals. In total, approximately 19.9% of all (n = 3840) stimulation pulses resulted in successful interruptions of SWD. Interruptions always occurred simultaneously in the cortex and thalamus, indicating that the effect of stimulation causing an interruption was not restricted to the cortex but involved the entire cortico–thalamo–cortical network.

A total of 343 *early* interruptions were identified across the three stimulation sessions (mean per rat: 57.2 ± 7.4 SEM). The distribution across stimulation intensities (in ascending order) was 103 (17.2 ± 2.6), 130 (21.7 ± 5.1), and 110 (18.3 ± 2.0). When distributed across the five stimulation intervals (in ascending order), the number of early interruptions was 105 (17.5 ± 3.6), 75 (12.5 ± 1.4), 67 (11.2 ± 1.4), 53 (8.8 ± 1.4), and 43 (7.2 ± 1.0). A comparable analysis for *late* interruptions revealed a total of 422 events (mean per rat: 70.3 ± 9.9). The distribution across stimulation intensities (ascending order) was 147 (24.5 ± 3.3), 136 (22.7 ± 5.4), and 139 (23.2 ± 3.6). Across the five stimulation intervals, the distribution was 64 (10.7 ± 5.6), 87 (14.5 ± 5.7), 85 (14.2 ± 5.7), 101 (16.8 ± 6.7), and 84 (14.0 ± 5.8), respectively.

A repeated-measures ANOVA with phase (early vs. late), stimulation intensity (three levels), and interval (five levels) as within-subject factors revealed only a marginally significant first-order interaction between phase and interval (p < 0.1). This effect was not explored further.

**Fig. 3:**
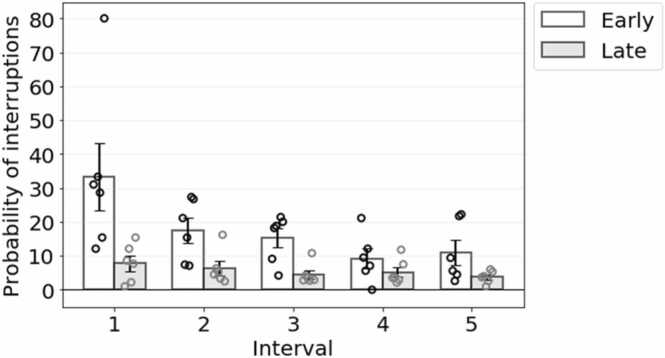
Probability as mean and sem of percentage of interruption for the early and late phase and for the five spike-wave intervals. The differences between the early and late phase were significant at interval 1, 2 and 3.

The lack of clear differences in susceptibility for the number of SWD interruptions may be due to an unequal number of stimulations at the five intervals and at the two SWD phases. In order to circumvent this issue, the percentages of successful interruptions were analysed. The percentage of interruptions was based on the percentage of interruptions per rat per interval per stimulation intensity. The mean percentage early SWD interruptions over the intervals, in ascending order, were: 33.4 (s.e.m. 10.0), 17.5 (3.7), 15.3 (2.8), 9.2 (2.9), and 11.0 (3.6), the percentages late interruptions were, again in ascending order: 7.8 (2.3), 6.3 (2.1), 4.5 (1.3), 5.2 (1.5), and 3.7 (0.7) and are depicted in Fig. 3.

A repeated measures ANOVA comparing the percentages of interruptions per interval and for the two phases (early-late) (stimulation Intensity was without an effect on the percentage interruptions) showed a main effect for phase (F=19.91, df 1,5, p < .01, ή^2^=.80), indicating that SWDs were more vulnerable for being interrupted in the early phase than in the late phase of an SWD. Next, a main effect for interval (F=3.65, df 4,20, p < .05, ή^2^=.42) and an interaction between phase and interval (F=3.23, df 4,20, p < .05, ή^2^=.39) were found. All effect sizes were large. The post-hoc tests following the significant phase x interval effect showed significant phase differences, that is, more interruptions during the first three intervals in the first second of an SWD only, and that the interval effect vanished in the late intervals and in the late phase.

### The influence of stimulation on the number and duration of SWDs

Stimulation could interrupt SWDs, and it did. On the other hand, stimulation could also induce ADs mimicking SWDs. This raised the question whether stimulation itself could have affected the number and mean duration of SWDs during the stimulation sessions. Therefore, the number and mean duration of SWDs as obtained during the stimulation sessions were compared with those during the baseline session. Neither the number of SWDs nor the mean duration of SWDs was affected by single pulse stimulation. The data can be found in Table 1.

**Table 1:** Features of the SWDs per experimental session.

**Experimental session**	**Total number of SWDs per hour**		**Duration SWDsin s**	
		**σ**		**σ**
**Baseline**	30.0	6.4	7.7	0.3
**20 μA stimulation**	36.4	5.0	7.9	0.7
**60 μA stimulation**	35.7	7.0	7.8	0.6
**100 μA stimulation**	32.3	4.8	7.8	0.8

= mean; σ = standard error of the mean

### Amplitudes of EEPs during the late phase of SWDs

**Fig. 4:**
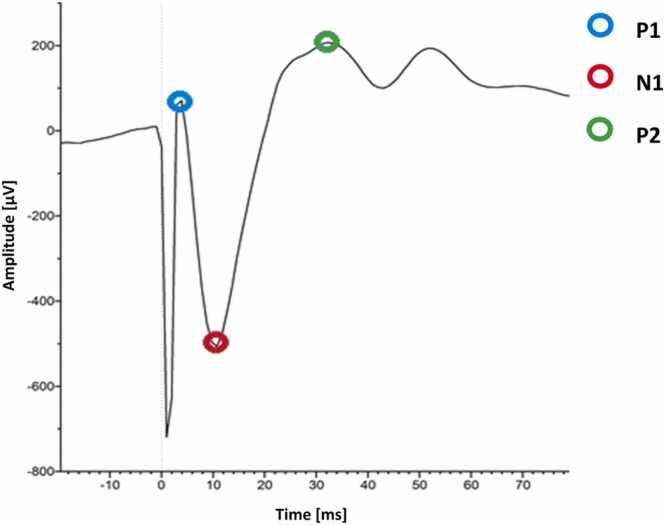
Grand grand average of the EEP per interval (n = 5) and per intensity (n = 3), for n = 6 subjects, representing the averaged response of a total of 900 individual stimuli equally divided over the five different intervals (1−2-3−4-5) and three different intensities (20–60-100 μA). Point of stimulation marked as Time = 0. The first negative peak is the stimulation artefact; the circles indicate the P1 (blue), N1 (red), and P2 (green). The first peak-peak amplitude is the amplitude of the artefact minus the amplitude of the P1, the difference between P1 and N1 and between N1 and P2 are the two other peak-peak amplitudes.

Grand average EEPs of the cortical EEG, made after the first second of SWD onset at non-interrupted stimulations in the five predefined intervals, showed three major components: P1, N1, and P2, see Fig. 4. Amplitude and latencies of the components per interval and per intensity are presented in Table 2.

**Table 2:** Amplitudes and latencies of P1, N1, and P2 of the EEPs by single pulse stimulation at 20, 60, and 100 μA in the somatosensory cortex of WAG/Rij rats (n = 6). Int = interval; = mean; σ = standard error of the mean. Amplitude in μV and latency in ms.

**20 μA**	**Amplitude P1**		**Amplitude N1**		**Amplitude P2**		**Latency P1**		**Latency N1**		**Latency P2**	
**Interval**	**[μV]**	**σ [μV]**	**[μV]**	**σ [μV]**	**[μV]**	**σ [μV]**	**[ms]**	**σ [ms]**	**[ms]**	**σ [ms]**	**[ms]**	**σ [ms]**
**Int. 1**	-114.51	47.31	-420.61	48.80	540.70	78.05	3.17	0.48	9.00	0.68	29.00	3.57
**Int. 2**	434.33	26.55	259.57	68.50	593.72	49.24	3.00	0.45	9.00	1.32	20.00	1.86
**Int. 3**	50.63	28.18	-345.57	63.50	-95.68	55.94	3.00	0.45	10.33	0.56	18.67	2.39
**Int. 4**	67.36	47.49	-299.94	75.60	21.63	64.50	3.00	0.45	10.33	0.76	19.17	2.10
**Int. 5**	134.33	42.36	-327.48	29.69	412.23	120.69	3.17	0.40	15.00	2.02	43.33	7.14

**60 μA**	**Amplitude P1**		**Amplitude N1**		**Amplitude P2**		**Latency P1**		**Latency N1**		**Latency P2**	
**Interval**	**[μV]**	**σ [μV]**	**[μV]**	**σ [μV]**	**[μV]**	**σ [μV]**	**[ms]**	**σ [ms]**	**[ms]**	**σ [ms]**	**[ms]**	**σ [ms]**
**Int. 1**	28.23	59.01	-722.16	154.89	698.62	70.81	3.50	0.56	10.50	0.43	29.83	4.47
**Int. 2**	530.70	40.15	-167.90	186.95	526.49	176.48	4.00	0.68	10.50	0.50	20.50	2.14
**Int. 3**	110.99	51.96	-752.41	184.96	-196.94	194.90	4.00	0.68	11.17	0.48	20.33	2.16
**Int. 4**	100.87	53.64	-733.09	158.97	-68.79	193.07	3.50	0.62	10.83	0.31	21.83	3.47
**Int. 5**	204.76	52.81	-680.14	120.44	-39.84	186.38	3.50	0.56	11.83	0.31	24.00	4.03

**100 μA**	**Amplitude P1**		**Amplitude N1**		**Amplitude P2**		**Latency P1**		**Latency N1**		**Latency P2**	
**Interval**	**[μV]**	**σ [μV]**	**[μV]**	**σ [μV]**	**[μV]**	**σ [μV]**	**[ms]**	**σ [ms]**	**[ms]**	**σ [ms]**	**[ms]**	**σ [ms]**
**Int. 1**	-13.77	81.32	-794.37	135.76	736.87	74.15	3.83	0.54	10.67	0.49	26.17	1.89
**Int. 2**	547.09	52.95	-290.78	142.40	837.15	55.00	3.83	0.54	12.17	1.20	26.50	2.20
**Int. 3**	90.25	68.29	-964.72	144.77	9.78	57.75	3.67	0.49	13.17	1.22	25.67	2.20
**Int. 4**	90.26	57.28	-876.30	130.22	186.30	61.44	3.67	0.49	12.50	1.12	25.33	2.01
**Int. 5**	214.23	70.72	-919.90	138.95	375.92	111.49	3.83	0.48	13.00	1.21	28.50	2.72

**100 μA < 1**^**st**^**sec.**	**Amplitude P1**		**Amplitude N1**		**Amplitude P2**		**Latency P1**		**Latency N1**		**Latency P2**	
**Interval**	**[μV]**	**σ [μV]**	**[μV]**	**σ [μV]**	**[μV]**	**σ [μV]**	**[ms]**	**σ [ms]**	**[ms]**	**σ [ms]**	**[ms]**	**σ [ms]**
**Int. 1**	-64.29	43.06	-819.56	134.35	696.99	120.32	4.50	0.43	11.00	0.37	27.67	2.29
**Int. 2**	363.72	83.04	-370.44	159.59	517.96	125.75	4.33	0.42	11.17	0.31	23.83	2.83
**Int. 3**	22.97	56.63	-971.36	134.48	-295.31	220.76	4.50	0.34	12.17	0.31	20.67	1.89
**Int. 4**	83.83	66.02	-897.91	136.54	-113.29	207.61	4.17	0.48	12.67	1.31	22.17	2.33
**Int. 5**	226.25	53.73	-847.56	156.32	144.95	197.60	4.33	0.42	12.17	1.05	25.50	3.40

**Fig. 5:**
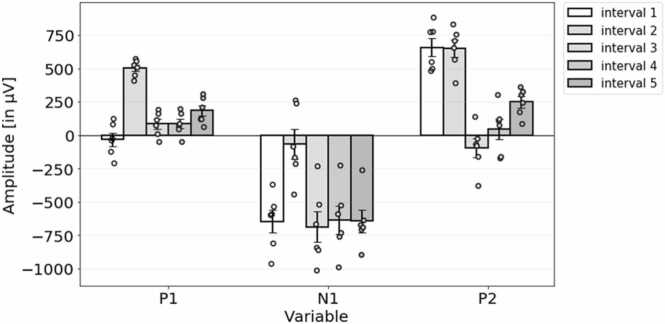
Mean and s.e.m. of the amplitude of P1, N1, and P2 per interval (intensity was pooled). Differences revealed by post hoc paired sample *t*-tests using Bonferroni correction were: P1: 1 < 2,4,5; 2 >1,3,4,5; 5 > 4; N1: 1,3,4,5 > 2; P2: 1,2 > 3,4,5.

Main and large interval effects were found for the amplitudes of P1 (F=96.78, df 4,20, p < .0001, ή^2^=.95), N1 (F=70.58, df 4,20, p < .0001, ή^2^=.93), and P2 (F=33.43, df 4,20, p < .0001, ή^2^=.87). The data are presented in Fig. 5. Post-hoc tests of the amplitude of P1 showed that it was significantly different (and in this case negative) in interval 1 compared to the positive amplitudes in intervals 2, 4, and 5; the amplitude of P1 at interval 2 exceeded the amplitude of all four other intervals, and stimulation at interval 5 evoked a larger P1 amplitude compared to interval 4 (p < .01). The post-hoc tests for the amplitude of the N1 showed much less negativity for interval 2 compared to all four other intervals. Last, the amplitude of P2 at intervals 1 and 2 exceeded significantly those of the intervals of 3, 4, and 5.

A main effect for intensity of stimulation was found for the amplitude of N1 (F=9.61, df 2,10, p < .01, ή=.66): a more negative amplitude was found for 100 μA compared to 20 μA (p < .01) while the 60 μA was in between the 20 and 100 μA. Also, the latency of P1 (F=6.01, df 2,10, p < .05, ή=.55) showed an intensity effect; the post-hoc tests showed that the latency of P1 was longer at 100 μA compared to 20 μA (p = .05).

An interaction between intensity and interval was found for the amplitude of P2 (F=2.46, df 8,40, p < .05, ή=.33). The post-hoc tests revealed that amplitude differences in the five intervals, as shown in Fig. 5, were more pronounced for the 100 μA than to the 20 μA condition.

### Peak-peak amplitudes of EEPs during SWDs at the late phase

The peak-peak amplitudes (artP1, P1N1, and N1P2) are presented in Table S1, and the amplitude of P1N1 and N1P2 in Fig S1. Main effects for interval were found for P1N1 (F= 13.46, df 4,20, p < .001 ή^2^=.73) and N1P2 (F=19.97, df 4,20, p < .001, ή^2^=.80). The post-hoc tests for P1N1 showed a smaller negative value at interval 2 compared to intervals 3, 4, and 5 (p < .01; p < .05; p < .05 respectively). Next, the amplitude of N1P2 at interval 1 exceeded those of intervals 2, 3, and 5 (all p’s < .05), on contrast, this amplitude at interval 2 exceeded that of interval 3 (p < .01). Main effects for intensity were found for the amplitudes of artP1 (F=10.45, df 2,10, p < .001, ή^2^=.68), P1N1 (F=12.54, df 2,10, p < .01, ή^2^=.71), and N1P2 (F=8.06, df 2,19, p < .01, ή^2^=.62). Post hoc tests showed that the amplitudes at 100 μA exceeded those of 20 μA for all three variables (all p’s < .05).

Interaction effects between interval and intensity for the amplitudes of P1N1 (F=2.31, df 8,40, p < .05, ή^2^=.32) and N1P2 (F=2.76, df 8,20, p < .05, ή^2^=.36) were found; subsequent post hoc tests for the P1N1 showed that the amplitude of 20 μA was smaller for intervals 1 and 2 compared to interval 5 (both p’s < .05), and interval 2 again smaller than interval 3 (p < .05). At 100 μA stimulation the amplitude at interval 2 was again smaller compared to those at intervals 3, 4, and 5 (p < .01; p < .05; p < .01, respectively)(Figure S2). The post-hoc tests for the N1P2 showed that the amplitude at 20 μA stimulation was larger in interval 1 compared to intervals 2 and 3 (p < .01; p < .05, respectively). At 60 μA stimulation, the amplitude at interval 2 exceeded that of interval 3 (p < .05), while at 100 μA stimulation, the amplitude of interval 1 exceeded that of interval 3 (p < .05)(Figure S3).

### Differences between non-REM sleep and SWD stimulation

**Fig. 6:**
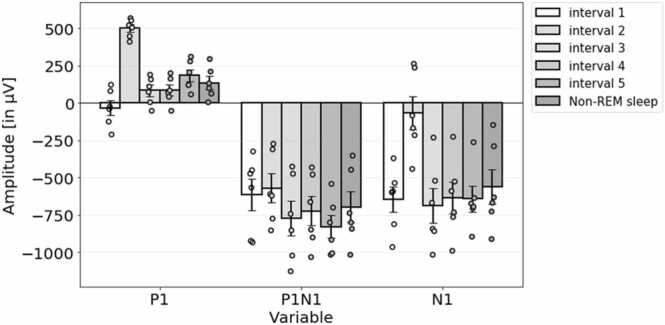
Non-REM sleep and Interval effects for the amplitudes of P1, P1N1, and N1 in μV. Bars show the mean amplitudes and their sem’s for the 100 µA conditions. Post-hoc tests comparing non-REM sleep with the five spike-wave intervals (1−5). P1: 2 > non-REM (6) > 1; P1N1: non-REM (6) > 2; N1: non-REM (6) > 2. For comparisons across the five intervals, see the legend of Fig. 5.

The EEP’s collected during non-REM sleep were compared to those of the five intervals. The data are presented in Fig. 6. Significant main effects were again found for the amplitude of the P1 (F=77.48, df 5,25, p < .001; ή^2^=.94), N1 (F=50.56, df 5,25, p < .001, ή^2^=.91) and P2 (F=26.38, df 5,25, p < .001, ή^2^=.84) and for the latencies of N1 (F=6.19, df 5,25, p < .001, ή^2^=.55) and P2 (F=8.06, df 5,25, p < .001; ή^2^=.62). Post-hoc tests now aimed to compare the non-REM sleep P1 amplitude with each of the P1 amplitudes of the five intervals showed a larger non-REM sleep P1 amplitude than at interval 1 (p < .05), but much smaller than at interval 2 (p < .01), while no differences were found with any of the wave-intervals. Post-hoc tests for the amplitude of the N1 showed a larger N1 during non-REM sleep compared to interval 2, and no differences with interval 1 and any of the wave intervals. The post-hoc tests for the amplitude of the P2 and for the latencies of N1 and P2 did not reveal differences between non-REM sleep and the other intervals. No differences were found between non-REM sleep stimulation and SWD intervals for the latencies of the P2 and the amplitudes of artP1 and N1P2. In summary, non-REM sleep stimulation differed from spike-stimulation in the amplitudes of P1, N1, and P1N1, while non-REM sleep stimulation resembled wave-stimulation for all the dependent variables tested. An overview of the differences found for the main effects of the amplitude-related variables for interval and during non-REM sleep is presented in Table S4.

### Early vs Late EEP’s: phase effects

**Fig. 7:**
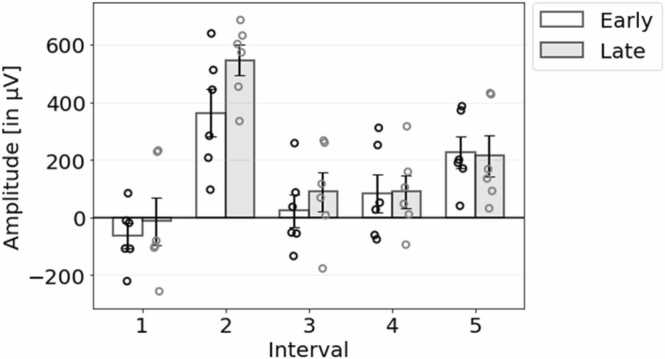
Interaction effect of interval (Early, within the first second after SWD onset (< 1st sec. with Late, after the first second after SWD onset (> 1st sec.) for the amplitude of P1. Bars show the mean and sem. Differences between the early and late P1 amplitude were revealed by post-hoc paired sample *t*-tests using Bonferroni correction and showed a larger P1 amplitude for interval 2 only.

The EEPs made at 100 μA within the first second after SWD onset (Early) were compared with those made one second after SWD onset (Late). Main effects for phase were found for the amplitude (F=10.45, df 1,5, p < .05, ή^2^=.67) and latency of P1 (F=12.27, df 1,5, p < .05, ή^2^=.71). The amplitude of P1 was smaller during the first second after SWD onset (p < .05), and its latency longer (p’s < .05). Besides the interval effect, already described in the 2nd section of the Results, an interaction between interval and phase was found for the amplitude of P1 and the amplitudes are illustrated in Fig. 7. Interval 2 evoked a larger (p < .05) amplitude of P1 when stimulation occurred > one second after SWD onset compared to stimulation within the first second after SWD onset (p < .05), showing that the phase effect was mainly due to the larger amplitude during the second interval.

## Discussion

The main goals of our research were to establish putative differences in cortical excitability between the two spike and three wave intervals of an SW complex through the probability of interruption of SWDs and through the EEP components elicited by local cortical electrical stimulation, and to compare these two excitability measures. Next, the EEP components were compared with those elicited during non-REM sleep in order to interpret the level of excitation/inhibition during the spike and waves. It was additionally investigated whether the phase of the SWD is relevant for both excitability measures. The response to single pulse stimulation in the vicinity of the focal region in the somatosensory cortex was measured, the region of the brain in which spontaneously occurring SWD originate. Clear differences in excitability were found dependent on the ascending and descending slope of the spike, during the wave, during the first vs the subsequent sec of an ongoing SWD, both regarding the probability of interruption and regarding various EEP components. A final result was that the excitability of the wave was comparable to that of non-REM sleep.

### Susceptibility to stimulus-induced interruption of SWDs

A major finding is that interruption of spike–wave discharges (SWDs) by single-pulse stimulation is consistently achievable across all five intervals, within and after the first second of an ongoing SWD, and all stimulation intensities. However, differences in interruption probability across stimulation conditions are confined to the first second; later, these differences are no longer observed. Single-pulse stimulation has similarly been shown to induce SWD interruption following cerebellar stimulation in mice (Eelkman Rooda et al., 2021).

The differential effects observed between the first second (early), and later phases of SWDs (Late) may reflect the incomplete network synchronization during the first second after onset. The hypothesized distinction between early and late interruption is grounded in prior findings demonstrating phase-dependent cortico-thalamic dynamics, including non-linear association measures and functional directional coupling (Meeren et al., 2002, Sysoeva et al., 2014, Sysoeva et al., 2016). Specifically, during the initial phase of an SWD, cortical activity predominantly drives thalamic activity, whereas after approximately one second, both structures alternately assume a leading role.

Importantly, early interruptions were preceded by up to four complete spike–wave cycles to ensure that a genuine SWD was present. Given that the interspike frequency of SWDs in WAG/Rij rats is approximately 8 Hz (range 7–10 Hz) (Drinkenburg et al., 1993, Sysoeva et al., 2014), these early interruptions in fact occurred at approximately 500 ms following SWD onset.

The number of interruptions appeared unaffected by stimulation intensity. If stimuli exert greater effects when large-scale neuronal synchronization is still developing—i.e.,before the emergence of the self-sustaining oscillatory excitatory dynamics that characterize SWDs beyond approximately one second—one would expect early-phase interruptions to be more effective at higher intensities. However, neither a main effect of intensity nor an interaction between phase (early vs. late) and intensity was observed. This absence of an intensity effect may reflect a ceiling effect, potentially occurring at intensities as low as 20 µA, suggesting that all applied intensities exceeded the threshold required to elicit a network response. The stimulation parameters used in the present study (20–100 µA, 1 ms pulse width) fall within the range commonly employed in intracranial rodent stimulation studies and are generally considered sufficient to modulate both local and large-scale network activity (van Heukelum et al., 2016, Grotemeyer et al., 2024, Abbasova et al., 2024).

Consistent with previous work, effective SWD interruption in this model, as well as in GAERS, appears to depend more critically on stimulation frequency and timing than on intensity (Lüttjohann and van Luijtelaar, 2013, Nelson et al., 2011). Notably, stimulation intensity did influence electrophysiological response characteristics: both N1 amplitude and peak-to-peak amplitudes were significantly larger following 100 µA compared to 20 µA stimulation. In contrast, the peak-to-peak amplitude of artP1 was exclusively determined by stimulation intensity and showed no modulation by interval, vigilance state (e.g., NREM sleep), or SWD phase (early vs. late). These findings indicate that the artP1 peak-to-peak measure primarily reflects the physical properties of the stimulus rather than the ongoing state of cortical network activity.

### Opposite effects within the spike intervals of the P1

Electrical stimulation paradigms are widely used to assess neuronal excitability. Stimulation-induced evoked potentials (EEPs) typically consist of a positive–negative–positive complex, with the P1 component serving as the primary marker of excitability. EEPs recorded from the somatosensory cortex are highly consistent across species and are sensitive to antiepileptogenic interventions (Allison and Hume, 1981, van Luijtelaar et al., 2013).

Robust main effects of interval were observed for all examined EEP variables. Most notably, the P1 component, occurring approximately 3–4 ms after stimulation and reflecting local cortical excitability, exhibited opposite patterns during the ascending and descending phases of the spike–wave complex. During the ascending phase of the spike (interval 1), the P1 amplitude was small and closely resembled responses observed during the wave intervals. In contrast, during the descending phase of the spike (interval 2), the P1 amplitude was markedly larger than in any other interval. The three wave intervals were largely comparable, although a gradual increase in P1 amplitude was observed toward the end of the wave.

The dissociation between spike intervals 1 and 2 supports the hypothesis of Depaulis et al. (2015) that the spike is not exclusively associated with excitation, but also involves inhibitory processes. Indeed, membrane potential dynamics during the spike reflect a complex interplay of excitatory and inhibitory activity (Chipaux et al., 2011, Polack et al., 2007). We propose that the reduced P1 amplitude during the ascending phase of the spike—comparable to that observed during the wave—reflects a state in which a large proportion of cortical neurons are already engaged in synchronous firing due to ongoing epileptic activity, leaving relatively few neurons available for recruitment by external stimulation. Conversely, the pronounced response during the descending phase suggests that the refractory period has largely subsided, allowing a greater number of cortical neurons to be recruited.

Depaulis et al. (2015) further proposed that the wave phase is not homogeneous, but exhibits intrinsic variability, partly attributable to depolarizing synaptic events in cortical pyramidal neurons (Charpier et al., 1999, Depaulis et al., 2015). Our working hypothesis was that stimulation during the spike would have limited impact, as many pyramidal neurons are either actively firing or in a refractory state, whereas stimulation during the wave would elicit relatively small responses due to dominant inhibitory influences, including activation of GABAergic interneurons. Consistent with this, the three wave intervals showed largely similar responses, with a modest increase toward the end of the wave. This late-wave increase may reflect the gradual termination of the inhibitory phase. As is well established, thalamic and cortical excitatory neurons become progressively less hyperpolarized before the activation of hyperpolarization-activated cyclic nucleotide–gated (HCN) channels and low-threshold T-type Ca²⁺ channels, which contribute to the burst firing that initiates the subsequent spike (van Luijtelaar and Zobeiri, 2014, Vickstrom et al., 2020). During the wave phase, cortical neurons are difficult to recruit by stimulation, as they are actively inhibited and hyperpolarized (Inoue et al., 1993, Kandel and Buzsáki, 1997, Pinault, 2003, Polack et al., 2007, Polack et al., 2009, Chipaux et al., 2011, Depaulis et al., 2015).”

The assignment of EEP components to the five intervals was based on the timing of stimulation within a given interval, with the P1 component serving as the primary marker of excitability. The subsequent components, N1 (latency about 12 ms) and P2 (latency about 25 ms), occur substantially later and may therefore fall into a different interval than the P1. For example, when the P1 occurs during the descending phase of the spike (estimated duration about 11 ms), the N1 may still occur within the same interval or shift into the subsequent interval, whereas the P2 will almost invariably occur in the next interval. Consequently, N1 and P2 are likely to be assigned to intervals that differ from that of the eliciting stimulus, which complicates their interpretation in relation to the phase of the spike–wave complex. This issue is less pronounced for the first two wave intervals, which have longer durations, but may still affect the interpretation of N1 and particularly P2 if the stimulation has occurred in the final wave interval. Notably, the N1 component occurring in the middle portion of the wave is minimally affected by this temporal overlap and shows amplitudes comparable to those observed in other wave intervals. Moreover, the differences observed between spike intervals for the P1 component are largely mirrored in the N1, supporting the robustness of these phase-dependent effects despite the temporal ambiguity.

The occurrence of N1 and P2 in intervals different from that of P1 has important implications for peak-to-peak measures of excitability. Specifically, peak-to-peak amplitudes of electrically evoked potentials—previously used to assess excitability during spike–wave discharges in Long–Evans rats (Shaw et al., 2006)—may reflect a mixture of spike- and wave-related activity. That is, the initial component of the evoked potential may occur during the spike, whereas subsequent components may fall within the wave, or vice versa, and this may invalidate the peak-peak amplitude during SWD as a marker of excitability. Despite this limitation, the spike–wave–modulated peak-to-peak amplitudes (P1–N1 and N1–P2; see Fig. S4) in the present study closely replicate the pattern reported by Shaw et al. (2006), namely reduced P1–N1 amplitudes during the spike relative to the wave. However, our interpretation of the P1 amplitude differs from that reported in a whisker stimulation study, which found predominantly larger sub- and suprathreshold intracellular responses—both indices of excitability—during the wave compared to the spike (Williams et al., 2020).

Several methodological differences likely account for this discrepancy. In the whisker stimulation study of Williams and co-authors, the first EEG component occurred at a latency of approximately 23 ms, and the 50 ms stimulation paradigm elicited both onset and offset responses. Moreover, the experiments were conducted in lightly anesthetized GAERS, and responses were strongly influenced by thalamic processing, particularly within the ventroposterior medial nucleus, the principal thalamocortical relay for somatosensory input. It is well established that thalamic gating plays a critical role in shaping cortical responses to peripheral stimulation (Coenen and Vendrik, 1972, Drinkenburg et al., 2003), and in that study, thalamic neuronal responses both preceded and closely mirrored cortical activity. This strongly suggests that cortical responses were largely determined by thalamic dynamics. All this hampers the comparison between the Williams et al. and our study, although we fully agree with Drinkenburg et al. (2003), Williams et al. (2020), and Shaw et al. (2006) that the transfer of sensory information during SWDs is still possible and comparable to non-REM sleep, that the animals are not deaf or blind during SWDs, and that incoming stimuli are differentially modulated during the spike than during the wave.

### A phase effect for P1

The interval effect on P1 amplitude was largely consistent across early (within the first second after SWD onset) and late phases (after the first second of SWD onset) of the SWD, although amplitudes were higher during the initial phase, primarily due to an increase in interval 2. The initiation phase corresponds to SWD onset, when cortical synchronization is still incomplete, and neurons are being progressively recruited into synchronous firing to sustain the discharge. It was therefore expected that stimulation responses would be larger during the first second after onset, before the establishment of a fully developed, self-sustaining oscillatory state in which a substantial proportion of cortical neurons participate in synchronized activity.

Contrary to this expectation, excitability was lower during the first second after SWD onset. The mechanisms underlying this finding remain unclear. One possibility is that, during the later phase, a larger population of neurons participates in precisely timed cortico-thalamic burst firing during the spike, compared to the early phase, when cortical recruitment and cortico-thalamic synchronization is still evolving. Qualitatively, we have often observed in the EEG signal a gradual increase in spike amplitude during the initial phase of SWDs, although this has not been formally quantified.

Previous work has shown that interspike frequency in WAG/Rij rats is higher during the first second of an SWD (10–13 Hz) and decreases thereafter (7–8 Hz) (Bosnyakova et al., 2007, Dolinina et al., 2022). However, how these temporal dynamics relate to the present findings on excitability remains unclear. Future studies employing intracellular recordings from multiple cortical neurons during early and late SWD phases may help elucidate the underlying mechanisms.

### The amount of inhibition during non-REM sleep

The amplitudes of the EEP during non-REM sleep were used as a reference for responses observed during spike–wave intervals. The primary EEP response (P1) during non-REM sleep closely resembled that observed during the wave phase of SWDs; no significant differences were found between wave intervals (3–5) and non-REM sleep. This suggests that the degree of cortical inhibition during the wave is comparable to that during non-REM sleep, irrespective of the up–down states characteristic of sleep oscillations. Accordingly, neuronal activity during the wave phase and averaged non-REM sleep may be functionally similar.

Previous evoked potential studies in WAG/Rij rats (e.g. Meeren et al., 2001) and auditory discrimination studies (Drinkenburg et al., 2003) have likewise concluded that afferent information processing during spike–wave activity remains possible and is broadly comparable to non-REM sleep, albeit reduced relative to wakefulness and REM sleep. During non-REM sleep, cortical neurons exhibit burst firing followed by periods of neuronal silence, although the non-REM sleep inhibitory phases are typically longer than during SWDs, consistent with the lower frequency of slow waves. Converging evidence indicates that both the wave component of the spike–wave complex and non-REM sleep are characterized by inhibitory synaptic processes leading to hyperpolarization and reduced neuronal firing (Inoue et al., 1993, Timofeev et al., 2001). Timofeev et al. (2001) described this inhibitory mechanism as disfacilitation, referring to a transient absence of synaptic input resulting in prolonged hyperpolarization of cortical neurons. Such states occur spontaneously during non-REM sleep due to reduced synaptic activity (Hirsch et al., 1983, Contreras et al., 1996, Timofeev et al., 2001). Given the similarity in EEP responses, it is plausible that comparable inhibitory processes underlie both non-REM sleep and the wave phase of SWDs, suggesting that disfacilitation mechanisms contribute to the generation of the wave component.

### Stimulation did neither affect the number of SWDs nor the duration of SWDs

The number and mean duration of SWDs were not affected by the stimulation protocol compared to control sessions without stimulation, indicating that repeated single-pulse stimulation did not induce neuroplastic changes in the mechanisms underlying SWD generation. These findings further suggest that the SWDs analyzed in the present study are representative of typical activity in this genetic absence epilepsy model. Plastic changes have been reported following frequent high-frequency stimulation of the substantia nigra in GAERS (Saillet et al., 2013).

**Two measures of excitability compared.** A major research question regarded whether the dynamics in cortical excitability during SWDs were related to the probability of interruptions of SWDs elicited by the same type of stimulation. The comparison between the two excitability measures showed that cortical stimulation during automatically detected SWDs interrupted ongoing SWD in the cortex and thalamus and is therefore affecting the whole network. The interruptions were sensitive for phase (more abortions in the first second than later), and showed an interaction between phase and interval, that is, the phase differences were restricted to interval 1,2,3, while the differences between intervals gradually decreased in the early phase and were non-existent in the late phase. The interruptions also lacked a stimulus intensity effect. In contrast, the EEP’s and in particular the P1 varied over the intervals both in the early and late phase with a sharp difference between interval 1 and 2, between 2 and 3,4,5 and between 4 and 5. The phase effect was restricted to interval 2, and the amplitudes of the EEP’s (N1 and peak-peak) showed an intensity of stimulation effect. Therefore, it is concluded that the probability of interruptions is dependent on the time passed since the onset of SWD, and it is easier to interrupt quickly after onset, probably due to the still not fully synchronized firing in the preferred interspike frequency. The cortical excitability, the P1, reflects a different phenomenon, although being elicited by the same stimulus. During the spike, its response seems to be dependent on the availability and readiness to fire of the cortical neurons: this is low during the ascending phase and very high during the descending phase of the spike. The low responsiveness during the wave might be due to cortical cells being hyperpolarized through a disfacilitation process. In all, the probability of SWD interruption does not depend on the local excitability as measured with EEPs, but depends on external stimuli, which may interrupt the oscillatory network activity. Both variables measure a different aspect of excitability. EEP indicates the local excitability, while the interruptions of SWDs are considered as a biomarker of network excitability.

## Limitations of the study

The sample size was modest, and interindividual variability in electrode positions may have influenced statistical outcomes and their interpretation. However, the observed effect sizes were large to very large, supporting the robustness of the reported effects.

SWD interruption was verified by visual inspection and is therefore inherently subject to some degree of observer bias. This was mitigated by applying strict and predefined criteria; ambiguous cases, such as transient reductions in spike amplitude smaller than one second, were excluded from the analysis.

### Electrode localization

Stimulation electrodes were positioned near the cortical focus, both within the cortex and in underlying subcortical structures, whereas EEG recordings were consistently obtained from the somatosensory cortex and thalamus. Consequently, stimulation likely affected multiple brain regions, while excitability was assessed at a single cortical site. Despite variability in electrode placement, responses were highly consistent across animals, and stimulation reliably induced SWD interruption irrespective of precise electrode location. These findings suggest that disruption of synchronized cortico-thalamo-cortical activity can be achieved by stimulating a range of brain regions, both within and outside the primary network, consistent with previous studies (Berenyi et al., 2012; Saillet et al., 2013; Blik, 2015; Lüttjohann and van Luijtelaar, 2015). Thus, interruption of SWDs does not appear to depend on stimulation of a specific anatomical locus, but rather on perturbation of the network as a whole. This further supports the notion that SWD interruption probability is not determined by local excitability, as measured here, but by the capacity of external stimuli to disrupt ongoing network synchronization.

## Conclusions

The cortical excitability in WAG/Rij rats varies markedly across the spike–wave cycle, as demonstrated by differences across the five predefined intervals. Notably, excitability also differs within the spike itself, whereas wave intervals show relatively homogeneous responses across EEP measures, with the exception of a modest increase toward the end of the wave. These findings support and extend the conclusions of Depaulis et al. (2015), indicating that the cellular mechanisms underlying the spike and the waves and their five different intervals differ substantially more than previously assumed, likely reflecting complex dynamic interactions between excitatory and inhibitory processes.

EEP responses during non-REM sleep closely resembled those observed during the wave phase, whereas clear differences were present relative to spike intervals, particularly interval 2, which exhibited the highest excitability. This indicates that excitability during spike interval 2 exceeds that of both spike interval 1 and all wave intervals.

In contrast, susceptibility to electrical stimulation, indexed by SWD interruption probability, was comparable across intervals despite pronounced differences in local excitability. This dissociation indicates that SWD interruption depends not on local cortical excitability, but on the ability of external stimuli to disrupt synchronized network activity. Moreover, mechanisms underlying SWDs differ between the initial and later phases, as reflected by the increased susceptibility to interruption during the first second after onset. The correct interpretation of peak-peak amplitudes in this type of research paradigm can be seriously questioned.

Overall, these findings highlight substantial heterogeneity in excitability across the spike–wave cycle and underscore the intrinsic complexity during the two spike and three wave components. Accordingly, a more fine-grained subdivision of the spike–wave cycle, beyond the conventional dichotomy of spike versus wave, is warranted.

## CRediT authorship contribution statement

**Bob de Ruijter:** Writing – original draft, Visualization, Project administration, Methodology, Investigation, Formal analysis, Data curation. **Gilles van Luijtelaar:** Writing – review & editing, Writing – original draft, Validation, Supervision, Methodology, Formal analysis, Data curation, Conceptualization.

## Ethics

The research was carried out in full compliance with EU Directive 2010/63/EU for animal experiments and approved by the Ethics Committee on Animal Experimentation of Radboud University, Nijmegen (RU-DEC).

## Declaration of Competing Interest

The authors declare that there is no conflict of interest.

## Footnotes

Appendix A Supplementary data associated with this article can be found in the online version at doi:10.1016/j.ibneur.2026.05.005.
