# Supplementary material for: Unraveling seizure interruptions: Excitability dynamics in spike-wave activity
Source: IBRO Neurosci Rep. 2026 May 22;20:808–19. doi: 10.1016/j.ibneur.2026.05.005 (PMC13253100; doi:10.1016/j.ibneur.2026.05.005)
Supplement: Supplementary file 1 — Supplementary material [file mmc1.docx]

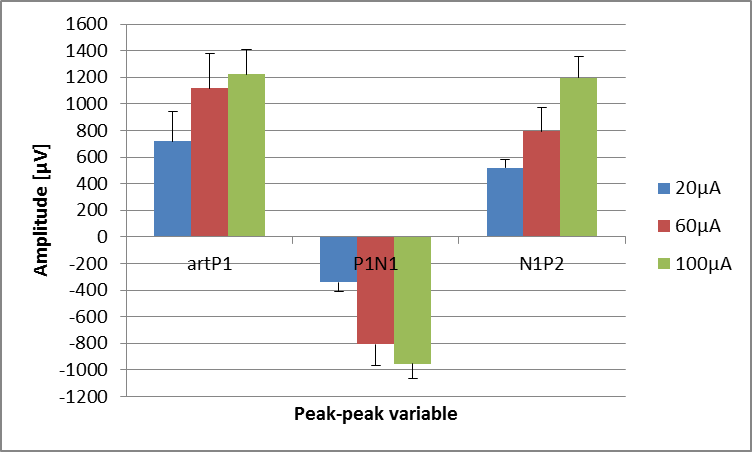


**Figure S1.** Mean and sem of the amplitude of the artP1, P1N1, and N1P2.Differences revealed by post hoc paired sample t-tests using Bonferroni correction showed that: artP1: 20 < 100 $\mu$A; P1N1: 20 < 100 $\mu$A; and N1P2: 20 < 100 $\mu$A.
