# Supplementary material for: Unraveling seizure interruptions: Excitability dynamics in spike-wave activity
Source: IBRO Neurosci Rep. 2026 May 22;20:808–19. doi: 10.1016/j.ibneur.2026.05.005 (PMC13253100; doi:10.1016/j.ibneur.2026.05.005)
Supplement: Supplementary file 2 — Supplementary material [file mmc2.docx]

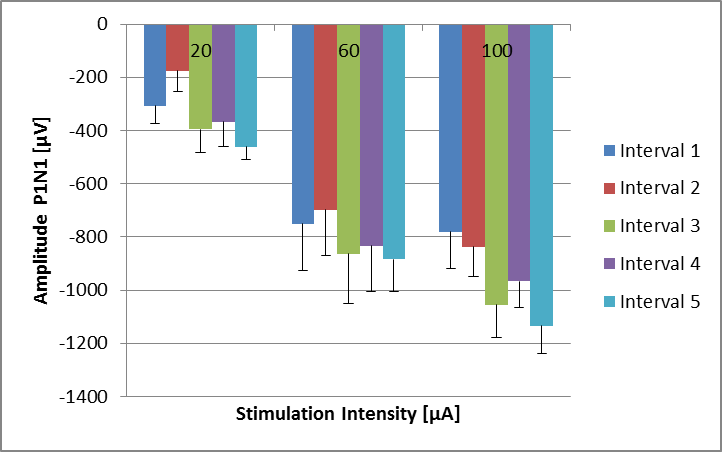


**Figure S2.** Mean and sem of the amplitudes of the peak-peak P1N1 per interval for the three intensities (left 20 µA, middle 60 µA, right 100µA). Interaction between intensity and interval: differences revealed by post hoc paired sample t-tests using Bonferroni correction showed that for 20 µA that 1,2 < 5; 2<3; for 60 µA : n.s.;
for 100 µA: 2 < 3,4,5.
