# Supplementary material for: Unraveling seizure interruptions: Excitability dynamics in spike-wave activity
Source: IBRO Neurosci Rep. 2026 May 22;20:808–19. doi: 10.1016/j.ibneur.2026.05.005 (PMC13253100; doi:10.1016/j.ibneur.2026.05.005)

**Figure S3.** Interaction effect of stimulation intensity and stimulation interval for the amplitude of the peak-peak value of N1P2. Bars show the average response of WAG/Rij rats (n=6), including the standard error of the mean. Differences revealed by post hoc paired sample t-tests using Bonferroni correction:

20: 1 >2,3.
60: 1 > 2,3,4,5; 2>3.
100: 1>3


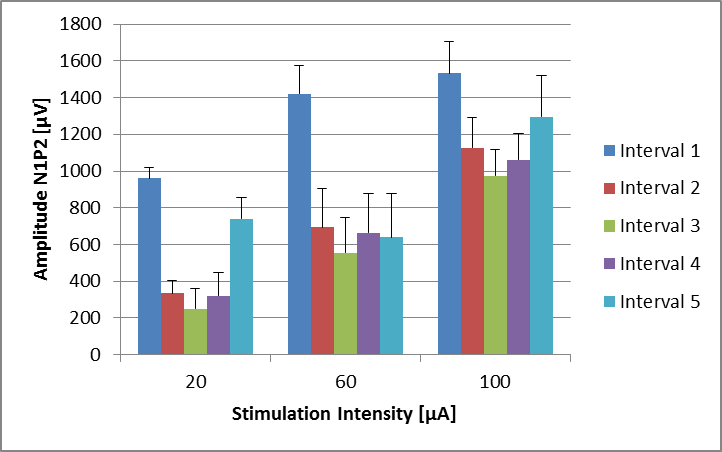

Supplement: Supplementary file 3 — Supplementary material [file mmc3.docx]
