# Supplementary material for: Unraveling seizure interruptions: Excitability dynamics in spike-wave activity
Source: IBRO Neurosci Rep. 2026 May 22;20:808–19. doi: 10.1016/j.ibneur.2026.05.005 (PMC13253100; doi:10.1016/j.ibneur.2026.05.005)
Supplement: Supplementary file 4 — Supplementary material [file mmc4.docx]

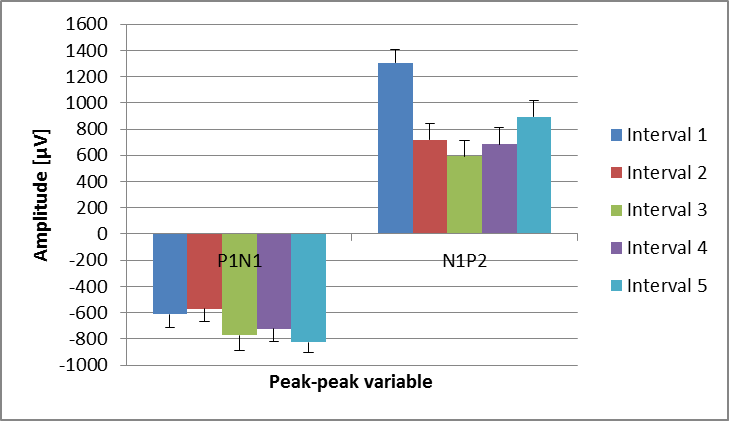


**Figure S4.** Main and sem of the peak-peak amplitude of the P1N1 and N1P2. Differences between intervals were revealed by post-hoc paired sample t-tests using Bonferroni correction, and showed that P1N1: 2 < 3, 4, 5; N1P2: 1 > 2, 3, 5; 2 > 3.
